# Human Brain Organoids Model Abnormal Prenatal Neural Development Induced by Thermal Stimulation

**DOI:** 10.1111/cpr.13777

**Published:** 2024-12-12

**Authors:** Lei Xu, Yufan Zhang, Xingyi Chen, Yuan Hong, Xu Zhang, Hao Hu, Xiao Han, Xiao Zou, Min Xu, Wanying Zhu, Yan Liu

**Affiliations:** ^1^ State Key Laboratory of Digital Medical Engineering, School of Biological Science and Medical Engineering, Department of Neurology, Affiliated Zhongda Hospital Southeast University Nanjing China; ^2^ Institute of Stem Cell and Neural Regeneration, School of Pharmacy Nanjing Medical University Nanjing China; ^3^ State Key Laboratory of Reproductive Medicine and Offspring Health Nanjing Medical University Nanjing China

**Keywords:** brain organoids, environmental stimulation, foetal brain development, maternal hyperthermia, neurodevelopmental disorders

## Abstract

The developing human foetal brain is sensitive to thermal stimulation during pregnancy. However, the mechanisms by which heat exposure affects human foetal brain development remain unclear, largely due to the lack of appropriate research models for studying thermal stimulation. To address this, we have developed a periodic heating model based on brain organoids derived from human pluripotent stem cells. The model recapitulated neurodevelopmental disruptions under prenatal heat exposure at the early stages, providing a paradigm for studying the altered neurodevelopment under environmental stimulation. Our study found that periodic heat exposure led to decreased size and impaired neural tube development in the brain organoids. Bulk RNA‐seq analysis revealed that the abnormal WNT signalling pathway and the reduction of G2/M progenitor cells might be involved in heat stimulation. Further investigation revealed increased neural differentiation and decreased proliferation under heat stimulation, indicating that periodic heat exposure might lead to abnormal brain development by altering key developmental processes. Hence, our model of periodically heating brain organoids provides a platform for modelling the effects of maternal fever on foetal brain development and could be extended to applications in neurodevelopmental disorders intervention.

## Graphical abstract

This study used periodically heated brain organoids derived from hPSCs to model prenatal heat exposure. It explored effects on size, neural tube development, gene expression, proliferation and differentiation, and their implications for neurodevelopmental disorders.

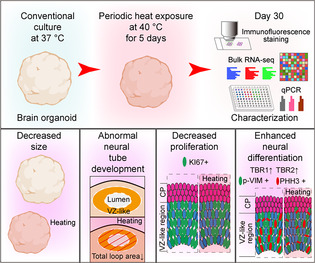

## 1 Introduction

Maternal fever during pregnancy may induce thermal stress in both the mother and foetus, potentially resulting in embryonic death, structural and functional defects or reduced growth. The developing brain during pregnancy is sensitive to thermal damage [1], and the specific phenotype depends on the increase and duration of maternal temperature, as well as the developmental stage of thermal damage [2]. Prospective and retrospective epidemiological investigations have underscored a potential association between early gestational hyperthermia and abnormal brain development [3, 4, 5, 6], including neural tube defects (NTDs) [2, 7, 8, 9, 10, 11], brain atrophy [12] and neuronal ectopia [5]. Therefore, heat exposure during pregnancy may lead to irreversible changes in the structure and function of the developing foetal brain.

Animal experiments across various species have demonstrated that prenatal exposure to heat stress is associated with a series of detrimental outcomes to the foetal brain [13, 14], including a decrease in brain weight [15, 16, 17], abnormal neurogenesis [18], microcephaly [17, 19], and NTDs [20, 21]. These findings highlight the critical impacts of maternal thermal conditions on neurodevelopmental trajectories in offspring. However, due to the lack of human brain development models, studies on the mechanism by which heat stress altered brain development are limited. It is difficult to quantitatively extrapolate from animals to humans due to species differences [22], even different sensitivities and thresholds for hyperthermia‐induced teratogenicity were shown within a species but different genotypes or strains [23, 24, 25, 26]. The demand to establish in vitro models of prenatal foetal brain development has become increasingly urgent [27].

Benefited by the development of human pluripotent stem cells (hPSCs), 3D‐cultured human brain organoids could model the endogenous microenvironment and biological processes of multifarious cells [28, 29, 30, 31]. Hence, brain organoids are widely used as in vitro models for toxicity tests including alcohol, nicotine and various viruses [29, 32, 33, 34, 35, 36], as well as for studying different neurological diseases [37, 38, 39, 40, 41, 42, 43, 44, 45, 46], especially in modelling diseases related to prenatal brain development [37, 38, 40, 44, 45]. Therefore, brain organoids may provide an opportunity for modelling the effects of hyperthermia during pregnancy on human foetal brain development in vitro.

Here, we proposed a strategy to use human brain organoids derived from hPSCs as developing foetal brain models to undergo periodic heat exposure at 40°C to model the influence of maternal fever on foetal brain development. We found that brain organoids indicated a diminution in size and impaired neural tube development after periodically heating. Subsequent bulk RNA‐seq unveiled upregulation of the WNT signalling pathway and neural differentiation transcription factors, coupled with a decline in G2/M progenitors in brain organoids after thermal stimulation. We thus confirmed that thermal damage led to attenuated proliferative activity within the ventricular zone (VZ) and augmented neural differentiation in the organoids. The proposed scheme of periodically heating brain organoids could offer a platform for research on mechanisms and drug screening in the study of foetal neurodevelopmental disorders (NDDs).

## 2 Materials and Methods

### 2.1 hPSCs Culture and Generation of Brain Organoids

Embryonic stem cells (ESCs) line H9 (WiCell agreement no. 16‐W0060) and induced pluripotent stem cells (iPSCs) line IMR90‐4 (WiCell agreement no. 17‐W0063) were used in the study [47, 48]. hPSCs were maintained in the culture plates coated with vitronectin (Thermo Fisher Scientific), and 2–3 mL of Essential 8 medium (Life Technologies) was added per well. When hPSCs reached 70%–80% confluency in the culture plate, they were treated with Dispase (1 U/mL, Gibco) for dissociation. DMEM/F12 (Thermo Fisher Scientific) was used to wash and dislodge the cells until all cells were detached. The cells were resuspended in neural induction medium (NIM) containing N2 supplement (Thermo Fisher Scientific), nonessential amino acids (MEM‐NEAA, Thermo Fisher Scientific), and DMEM/F12 (Thermo Fisher Scientific), and transferred to culture flasks for suspension culture. The cells self‐organised to form embryoid bodies (EBs) designated as day 0. EBs were cultured in NIM containing 2 μM SB431542 (Tocris) and 2 μM DMH1 (Tocris) from day 1 to day 7. At day 7, EBs were adhered in the culture plates added NIM with 10% FBS. After 8–10 h, the medium was replaced with FBS‐free NIM, and half‐medium change was performed every other day. At day 10, rosette‐like structures were observed under the microscope. At day 16, the adhered rosettes were disaggregated to form brain organoids by 1 mL pipette. The brain organoids were transferred to a culture flask for suspension culture and continuously cultured until day 30.

### 2.2 Periodic Heat Exposure Scheme of Brain Organoids

Two CO_2_ incubators were prepared in advance. One was set to 37°C as a normal environment to model normal body temperature, and the other one was set to 40°C as a heat exposure environment to model maternal fever. As the periodic heat exposure scheme, brain organoids could be transferred from a 37°C incubator to a 40°C incubator to model maternal periodic fever at 09:30–10:30 in the morning and 15:30–16:30 in the afternoon. During the neural tube formation stage (day 10–14), the periodic heat exposure scheme was applied to brain organoids as the D10‐14 heating group. Likewise, during the critical stage of neural tube development (day 20–24), the periodic heat exposure scheme was applied to brain organoids as the D20‐24 heating group. Brain organoids that had not undergone periodic heat exposure were used as the control. The alignment of the D10‐14 heating phase with neural tube formation and the D20‐24 heating phase with critical subsequent developmental stages provided a temporal framework for assessing the impact of thermal exposure.

### 2.3 Frozen Section and Immunofluorescence Staining of Brain Organoids

At day 30, brain organoids were fixed with 4% paraformaldehyde for 2–4 h in 1.5 mL Eppendorf tubes. The organoids were washed with PBS for 10 min 3 times, and 20% sucrose solution was added for dehydration of brain organoids at 4°C overnight. When the organoids settled to the bottom of the tube, the 20% sucrose solution was replaced with 30% sucrose solution for further dehydration until the organoids settled to the bottom of the tube again. Brain organoids were embedded in OCT compound and frozen in the cryostat (Leica). Once organoids were frozen, they were cut into sections with a thickness of 20 μm. The sections were attached to polylysine‐coated adhesive slides and stored at −20°C.

The sections were washed for 10 min 3 times with PBS to remove OCT compound, and then blocked and permeabilized in PBS containing 1% Triton (Bio‐Link) and 5% donkey serum (MilliporeSigma) for 1 h. The sections were incubated in primary antibodies diluted in PBS containing 0.2% Triton (Bio‐Link) and 5% donkey serum (MilliporeSigma) at 4°C overnight. On the next day, the sections were washed for 10 min 3 times with PBS to remove primary antibodies, and then incubated in secondary antibodies diluted in PBS containing 5% donkey serum for 1 h at 20°C. The sections were then washed for 10 min 3 times with PBS to remove secondary antibodies. Finally, coverslips were mounted with antifade mounting medium (Southern‐biotech) for fluorescence imaging.

### 2.4 Tissue Clearing and 3D Reconstruction of Brain Organoids

Three groups of brain organoids derived from ESCs (H9) were collected in 1.5 mL Eppendorf tubes at day 30. The organoids were fixed with 4% paraformaldehyde for 1 h, followed by three washes with PBS for 8 min each. They were then permeabilized with 1% Triton X‐100 for 1 h and blocked with 5% donkey serum for 4 h. After incubation with primary antibodies (PKC‐λ and SOX2) diluted in a solution containing 0.2% Triton X‐100 and 5% donkey serum overnight, the organoids were washed three times with PBS for 10 min each. Subsequently, they were incubated with secondary antibodies diluted in 5% donkey serum for 4 h, followed by another three washes with PBS for 8 min each. The organoids were then dehydrated stepwise with increasing concentrations of tert‐butanol (30%, 50%, 70%, 80%, 90%, 96% and 100%) for 1 h at each step. Finally, the organoids were immersed in BABB‐D4 solution (20% diphenyl ether, 26.7% benzyl alcohol and 53.3% benzyl benzoate) and imaged using confocal microscopy (Zeiss LSM 800) to obtain layer scan images. These images were imported into Imaris software for 3D reconstruction.

### 2.5 Bulk RNA Sequencing (RNA‐Seq) and Bioinformatics Analysis

Three groups of brain organoids derived from ESCs (H9) contained at least 15 organoids in each group. Total RNA of brain organoids was extracted using a TRIzol reagent kit (Invitrogen, Thermo Fisher Scientific), and HiSeq 4000 sequencing platform (Illumina) was used for library construction and high‐throughput RNA Seq. The gene abundances were calculated and normalised to transcripts per million (TPM). Differentially expressed genes (DEGs) between the D10‐14 heating and control and DEGs between the D20‐14 heating and control were determined by using the DESeq2 (55) package (version 1.30.0). An adjusted *p*‐value < 0.05 and an absolute log_2_ (fold change) ≥ 1 were set as the cut‐off criterion. The websites Enrichr and String were used for Gene Ontology (GO) analysis, Kyoto Encyclopedia of Genes and Genomes (KEGG) analysis and Protein–Protein Interaction (PPI) networks. Gene Set Enrichment Analysis (GSEA) was obtained through the MSigDB database. The genes related to multiple diseases in DisGeNET were called, and a heatmap was drawn based on the gene disease correlation score in DisGeNET. Spearman regression analysis was performed on the normalised average expression of brain organoids transcriptome data from different diseases, and the normalised average expression was visualised after spearman regression analysis.

### 2.6 Quantitative Real‐Time Polymerase Chain Reaction (qPCR)

Total RNA of brain organoids was extracted by using a TRIzol kit (Thermo Fisher Scientific). 1 mg total RNA from each sample was reverse transcribed into cDNA, and then the SuperScript III first strand synthesis system (Thermo Fisher Scientific) was used to perform qPCR. GAPDH was applied as housekeeping gene. The primers used for qPCR were as follows: KI67 forward primer, GAC CTC AAA CTG GCT CCT AAT G and reverse primer, GCT GCC AGA TAG AGT CAG AAA G; PAX6 forward primer, GCG GAA GCT GCA AAG AAA TAG, and reverse primer, GGG CAA ACA CAT CTG GAT AAT G; SOX2 forward primer, CAT CAC CCA CAG CAA ATG AC, and reverse primer, GAA GTC CAG GAT CTC TCT CAT AAA; TBR1 forward primer, GGT TTC CCA CTT CTC CTC AAT, and reverse primer, GCC TAT GAA CAG ACA CCT ATC C; TBR2 forward primer, GTG GCA AAG CCG ACA ATA AC, and reverse primer, CCG AAT GAA ATC TCC TGT CTC A; GAPDH forward primer, CGC TGA GTA CGT CGT GGA GTC, and reverse primer, GCT GAT GAT CTT CAG GCT GTT GTC.

### 2.7 Statistical Analysis

The bright‐field images of the organoids were captured and preserved daily using an optical microscope. These images were then opened in Fiji (ImageJ, v1.51s), and polygon selections were used to outline the edges of the organoids, thereby measuring their areas and perimeters. Hoechst staining was used as a marker to count the total number of cells, and cells in the fluorescence were counted and analysed by Fiji (ImageJ, v1.51s). All data was presented as mean ± SEM, and each group of data included three or more randomised independent experiments. Statistical analyses were performed using one‐way ANOVA or Chi square test. *p* < 0.05 was considered statistically significant.

## 3 Results

### 3.1 Brain Organoids Exhibited Decreased Size After Periodically Heating

**FIGURE 1:**
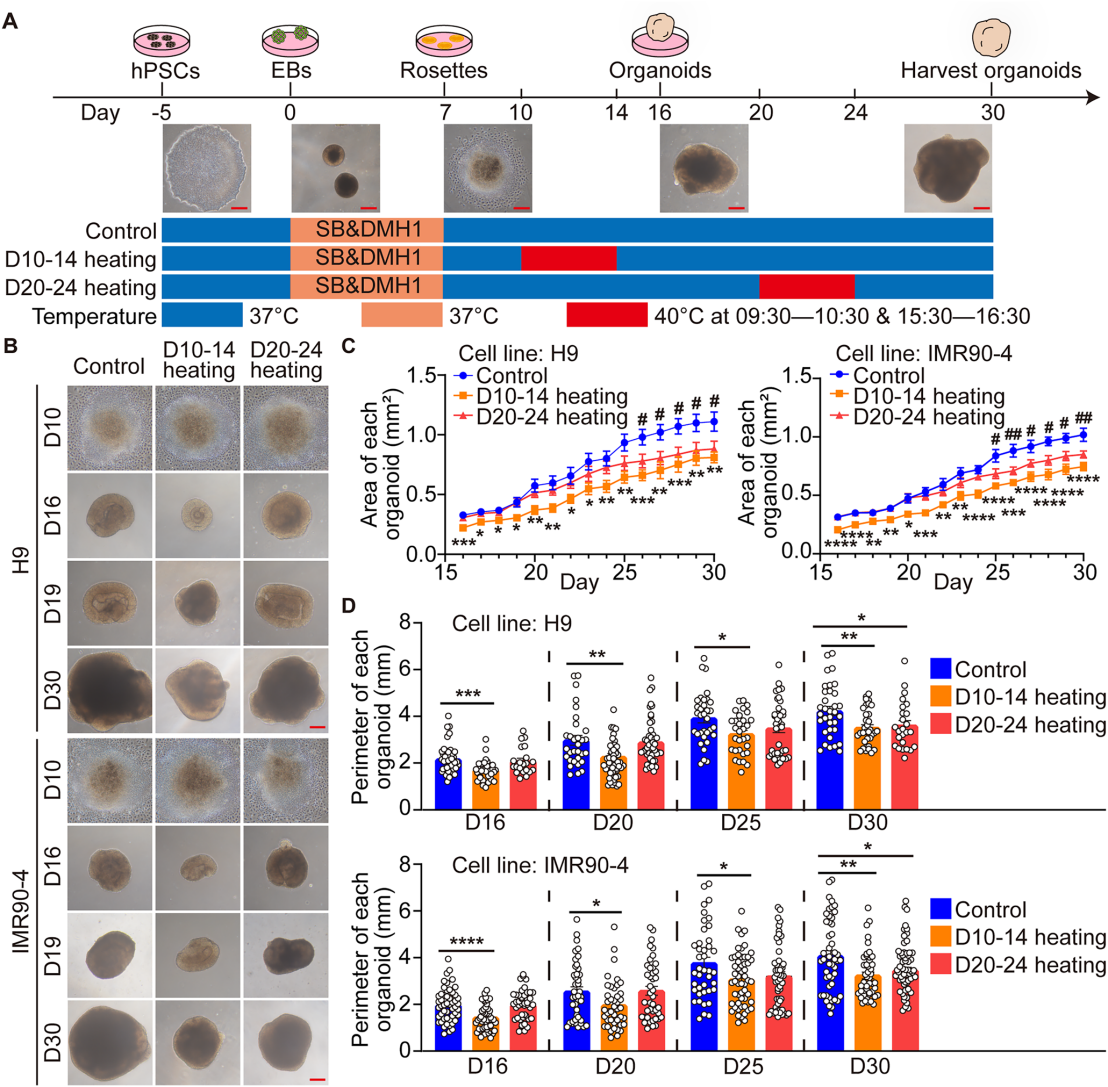
The size of brain organoids decreased after periodically heating. (A) The differentiation process of brain organoids and schematic diagram of periodic heat exposure. Scale bars, 250 μm. (B) Representative bright field images of brain organoids derived from ESCs and iPSCs in three groups of the D10‐14 heating, D20‐24 heating and control at day 10, 16, 19 and 30. Scale bars, 250 μm. (C) The line graph of the average area of brain organoids in three groups increasing over time under the bright field, *: Significant differences between the D10‐14 heating and the control group, #: Significant differences between the D20‐24 heating and the control group (H9, Control: *n* = 37, D10‐14 heating: *n* = 37, D20‐24 heating: *n* = 25; IMR90‐4, Control: *n* = 49, D10‐14 heating: *n* = 48, D20‐24 heating: *n* = 55). (D) The histogram of the perimeters of brain organoids in three groups at different time under the bright field (H9, Control: *n* = 37, D10‐14 heating: *n* = 37, D20‐24 heating: *n* = 25; IMR90‐4, Control: *n* = 49, D10‐14 heating: *n* = 48, D20‐24 heating: *n* = 55). Each data point and relative error bar corresponds to the average and standard deviation of at least 60 measured samples for at least three independent experiments. Statistical analysis was performed by ANOVA. *p < 0.05, **p < 0.01, ***p < 0.001, ****p < 0.0001.

To model human foetal brain under thermal exposure, we generated brain organoids from hPSCs including ESCs (H9) and iPSCs (IMR90‐4) by modifying the protocol of brain organoid described in the previous report [44, 45] (Figure 1A). During the continuous culture until day 30 for harvesting organoids, the brain organoids demonstrated a time‐dependent increase in volume (Figure 1B,C). After undergoing periodically heating, a discernible reduction in the area (H9, Control: 1.11 mm^2^, D10‐14 heating: 0.81 mm^2^, D20‐24 heating: 0.88 mm^2^; IMR90‐4, Control: 1.02 mm^2^, D10‐14 heating: 0.75 mm^2^, D20‐24 heating: 0.85 mm^2^) and perimeters (H9, Control: 4.23 mm, D10‐14 heating: 3.51 mm, D20‐24 heating: 3.61 mm; IMR90‐4, Control: 4.07 mm, D10‐14 heating: 3.24 mm, D20‐24 heating: 3.47 mm) of the organoids was observed at day 30 (Figure 1B–D). Compared with the control group, the D10‐14 heating group exhibited size diminution from the day of transitioning from rosettes adherent culture to resuspension culture (day 16), which persisted up to day 30 (Figure 1B–D). Correspondingly, the D20‐24 heating group showed a significant reduction in both the area and circumference of organoids under the bright‐field imaging by day 26, 6 days into the heating exposure (Figure 1B–D).

Taken together, brain organoids that underwent periodic heating treatment demonstrated a decrease in size, which was sustained until the harvesting of organoids.

### 3.2 Periodic Thermal Exposure Led to Abnormal Neural Tube Development in Brain Organoids

**FIGURE 2:**
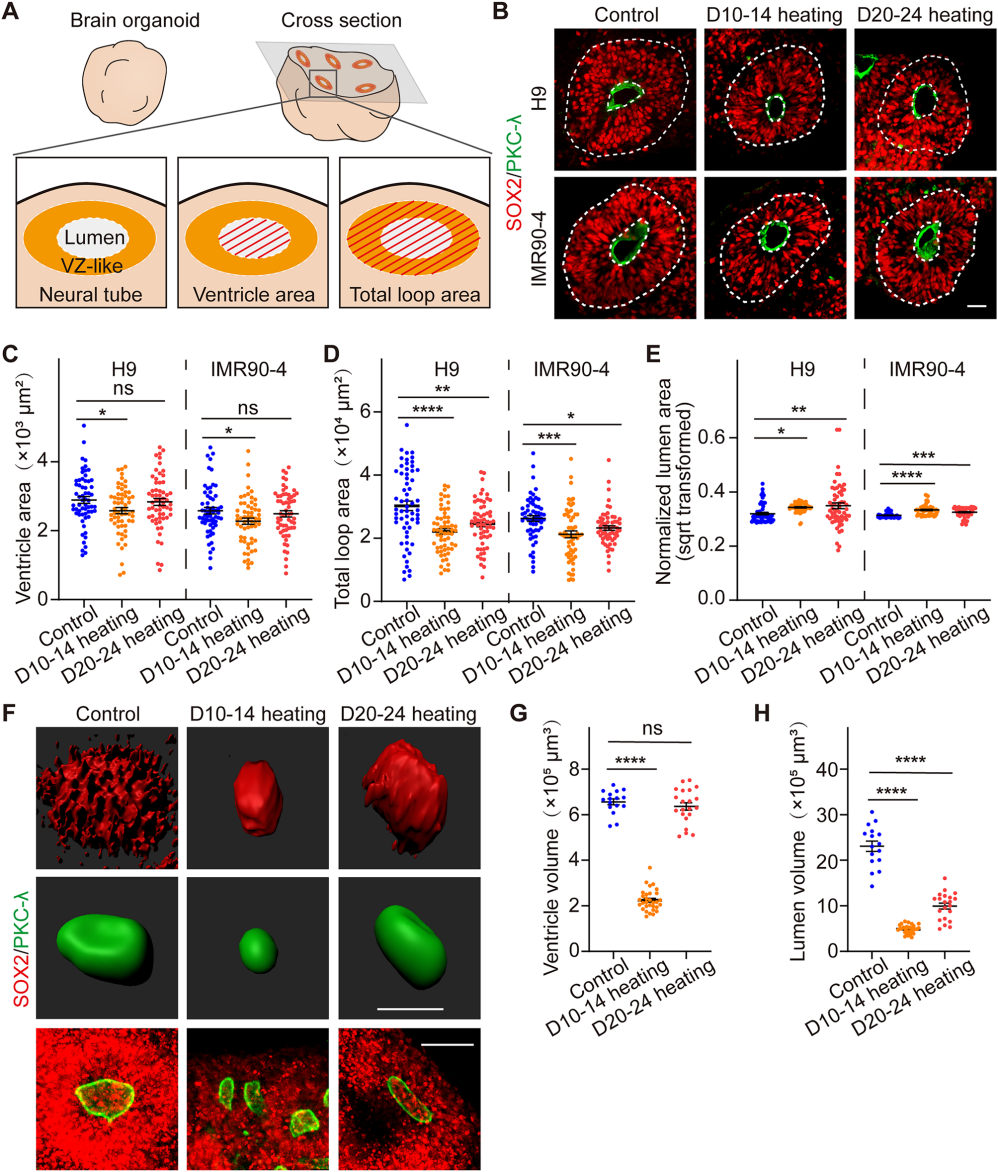
Brain organoids exhibited abnormal neural tube development after periodically heating at day 30. (A) Schematic diagram of statistical indicators for neural tube structure. (B) Representative immunofluorescent staining of the apical surface's marker PKC‐λ and neural progenitors' marker SOX2 in brain organoids derived from ESCs and iPSCs in three groups of the D10‐14 heating, D20‐24 heating and control at day 30, Scale bars, 50 μm. (C, D) Ventricle area and total loop area of brain organoids in three groups (H9, Control: *n* = 60, D10‐14 heating: *n* = 60, D20‐24 heating: *n* = 60; IMR90‐4, Control: *n* = 60, D10‐14 heating: *n* = 60, D20‐24 heating: *n* = 60). (E) Normalised lumen size (ventricle area/total loop area, square root transformed) of brain organoids in three groups (H9, Control: *n* = 60, D10‐14 heating: *n* = 60, D20‐24 heating: *n* = 60; IMR90‐4, Control: *n* = 60, D10‐14 heating: *n* = 60, D20‐24 heating: *n* = 60). (F–H) Representative immunofluorescent staining, 3D reconstruction and volumes of the ventricle and lumen regions of organoids derived from ESCs in three groups of the D10‐14 heating, D20‐24 heating and control at day 30 (Control: *n* = 16, D10‐14 heating: *n* = 31, D20‐24 heating: *n* = 21). Scale bars, 50 μm. Each data point and relative error bar corresponds to the average and standard deviation of at least 60 measured samples for at least three independent experiments. Statistical analysis was performed by ANOVA. *p < 0.05, **p < 0.01, ***p < 0.001, ****p < 0.0001.

Both clinical and animal studies suggested a possible relevance between maternal thermal damage during pregnancy and foetal NTDs [2, 7, 8, 9, 10, 11, 20, 21]. Indeed, we observed abnormal structure of the neuroepithelial loop in the organoids derived from ESCs or iPSCs after heat exposure at day 30, including a reduction in the ventricle area of D10‐14 heating organoids, as well as a decrease in the total loop area of D10‐14 and D20‐24 heating organoids [44] (Figure 2). PKC‐λ was utilised to define the area of each apical surface, and SOX2 was employed to define the VZ‐like area as the total loop area of the organoids by immunofluorescence staining (Figure 2A,B). We observed a decrease in the ventricle area of organoids after D10‐14 heating treatment (H9, Control: 2.89 × 10^3^ μm^2^, D10‐14 heating: 2.58 × 10^3^ μm^2^, D20‐24 heating: 2.83 × 10^3^ μm^2^; IMR90‐4, Control: 2.58 × 10^3^ μm^2^, D10‐14 heating: 2.27 × 10^3^ μm^2^, D20‐24 heating: 2.49 × 10^3^ μm^2^), whereas the D20‐24 heating exposure did not exhibit a statistically significant difference on the ventricle area of organoids (Figure 2C). Such findings suggest that the area of the apical surface is susceptible to damage under thermal stimulation during the phase of neural tube formation.

Upon assessing the total loop area across the six groups of organoids (H9, Control: 3.03 × 10^4^ μm^2^, D10‐14 heating: 2.22 × 10^4^ μm^2^, D20‐24 heating: 2.46 × 10^4^ μm^2^; IMR90‐4, Control: 2.63 × 10^4^ μm^2^, D10‐14 heating: 2.13 × 10^4^ μm^2^, D20‐24 heating: 2.33 × 10^4^ μm^2^), both D10‐14 and D20‐24 heating exposures were found to significantly reduce the total loop area of organoids (Figure 2D). The results indicated that thermal damage could lead to abnormal neural tube development. Moreover, we quantified the normalised lumen area (ventricle area/total loop area, square root transformed) of six groups of organoids [49, 50], and an increase in the relative lumen area of organoids was observed after periodically heating (Figure 2E), reflecting abnormal neural tube development after periodic heat exposure.

Furthermore, we analysed the volumes of the ventricle and lumen regions of organoids derived from ESCs (H9) using tissue clearing technology for 3D reconstruction to confirm the defect in neural tube development after periodic heating at day 30 (Figure 2F). Consistent with the area analysis, both D10‐14 and D20‐24 heating organoids showed a reduction in lumen volume (Control: 23.07 × 10^5^ μm^3^, D10‐14 heating: 4.86 × 10^5^ μm^3^, D20‐24 heating: 9.95 × 10^5^ μm^3^) (Figure 2G). Additionally, D10‐14 heating organoids also exhibited a decrease in ventricle volume (Control: 6.57 × 10^5^ μm^3^, D10‐14 heating: 2.24 × 10^5^ μm^3^, D20‐24 heating: 6.37 × 10^5^ μm^3^) (Figure 2H).

In conclusion, our results indicated that periodic thermal damage could induce abnormal neural tube development in brain organoids, posing significant implications for the study of hyperthermia‐induced teratogenic effects during embryonic neural development.

### 3.3 Reduction of G2/M Progenitors and Abnormal Alterations of NDDs‐Related Genes in Brain Organoids Were Induced by Periodically Heating

**FIGURE 3:**
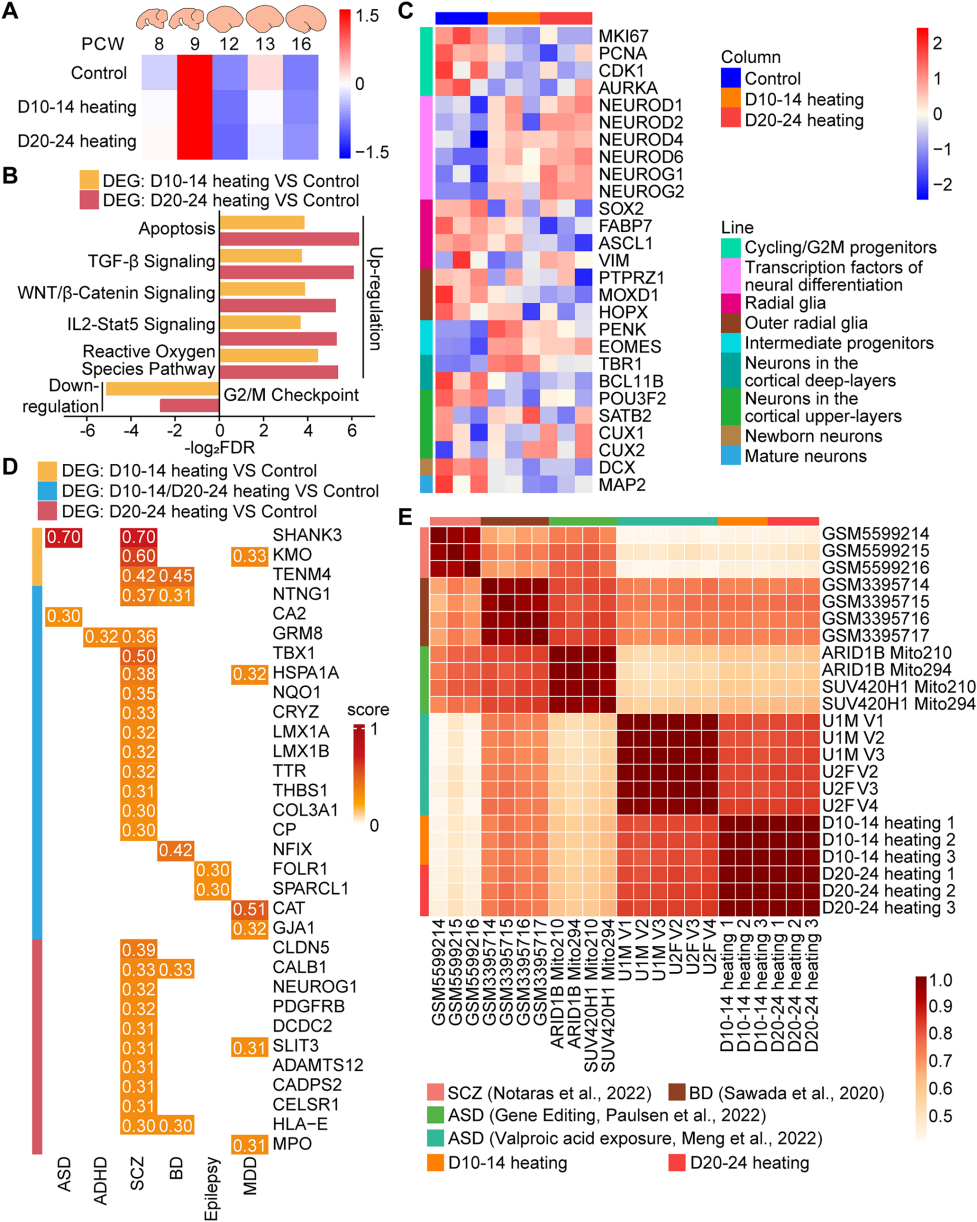
Brain organoids derived from ESCs revealed transcriptomic changes after periodically heating at day 30. (A) Transcriptome correlation between three groups of brain organoids at day 30 and developing human brain samples from the BrainSpan project (PCW 8–16). The mean Spearman's correlation coefficients. (B) Histogram of GSEA analysis based on DEGs. (C) Heatmap illustrating the expression level of DEGs enriched in different cell types, which is normalised by row. (D) Heatmap illustrating the correlation score between DEGs and NDDs called from DisGeNET. (E) Correlation heatmap reflecting the similarity between brain organoids after periodically heating and brain organoids derived from different NDDs.

To further explore the impact of periodically heating on the development of brain organoids, we profiled three groups of brain organoids derived from ESCs (H9) at day 30 utilising Bulk RNA‐seq. The comparative analysis of our sequencing data with the human brain post‐conceptional weeks (PCW) 8–16 data from the BrainSpan database indicated that all three groups of organoids exhibited a closer resemblance to the 9 PCW human brain (Figure 3A). Principal component analysis (PCA) showed significant transcriptomic differences in organoids among the D10‐14 heating, D20‐24 heating and control groups (Figure S1A), denoting significant changes in gene expression patterns. Relative to the control, the D10‐14 heating group had 156 up‐regulated genes and 15 down‐regulated genes, while the D20‐24 heating group had 282 up‐regulated genes and 12 down‐regulated genes (Figure S1B,C). Of note, 140 DEGs from the D10‐14 heating group exhibited consistent upregulation when compared to the DEGs of the D20‐24 heating group relative to the control (Figure S1D).

GO analysis of the 140 concordantly upregulated DEGs identified significant enrichment in processes and pathways pivotal for organoid development, including the WNT, BMP and TGF‐β signalling pathways, and mechanisms governing neural differentiation (Figure S1F). Complementary KEGG pathway and PPI analyses reaffirmed the activation of WNT (D10‐14 heating: 35.13‐fold increase in WNT1, 15.45‐fold increase in WNT3A, 7.45‐fold increase in WNT5A, and 16.91‐fold increase in WNT8B; D20‐24 heating: 44.65‐fold increase in WNT1, 37.14‐fold increase in WNT3A, 15.26‐fold increase in WNT5A, and 26.04‐fold increase in WNT8B) and TGF‐β (D10‐14 heating: 15.30‐fold increase in CDKN2B, 4.39‐fold increase in BAMBI, 2.11‐fold increase in ID1, and 2.82‐fold increase in ID3; D20‐24 heating: 31.27‐fold increase in CDKN2B, 9.54‐fold increase in BAMBI, 3.63‐fold increase in ID1, and 4.29‐fold increase in ID3) pathways as a consequence of periodically heating (Figure S1G,H). As a core gene in the PPI analysis, it is worth noting that WNT5A is a canonical member of the secreted WNT protein family, known for its role in WNT/PCP signalling and critical for neural tube closure through mechanisms such as convergent extension [51, 52, 53, 54, 55]. We also performed GO and KEGG analysis on non‐overlapping genes in two groups of DEGs, and the results still showed up‐regulation of the WNT pathway (Figure S1I).

We further analysed 140 overlapping DEGs by GSEA and results showed the up‐regulation of apoptosis, TGF‐β Signalling, WNT/β‐Catenin Signalling, IL2‐Stat5 Signalling and reactive oxygen species pathway (Figures 3B and S2). We also found down‐regulation of G2/M checkpoint, which might indicate the impact of periodically heating on the cell cycle within the organoids (Figures 3B and S2). Expanding upon these findings, we evaluated the expression of canonical markers of G2/M progenitors alongside transcription factors implicated in neural differentiation across 9 samples from three groups organoids, which might suggest disrupted proliferation and abnormal neural differentiation in the organoids after heat exposure (Figure 3C). Additionally, we further investigated the relative expression of typical biomarkers of different cell types in brain organoids. The findings indicated a decrease in radial glia and outer radial glia, an increase of intermediate progenitors (D10‐14 heating: 2.17‐fold increase in PENK, and 4.60‐fold increase in EOMES; D20‐24 heating: 1.54‐fold increase in PENK, and 4.18‐fold increase in EOMES) and the disorder of cortical stratification (Figure 3C).

Considering the established correlation between prenatal fever and a heightened risk for NDDs [56, 57, 58, 59, 60], we intersected the set of DEGs with genes implicated in autism spectrum disorder (ASD), attention deficit hyperactivity disorder (ADHD), schizophrenia (Scz), bipolar disorder (BD), epilepsy, and major depressive disorder (MDD) as delineated by DisGeNET. Constructing a heatmap based on the correlation scores between DEG and disease, we found that periodic heat exposure might be associated with NDDs (Figure 3D). We also intersected DEGs with 611 genes related to NDDs [61], and identified a set of NDDs‐related genes that might be associated with periodically heating (Figure S3A,B). Moreover, we performed Spearman regression analysis on our sequencing data and previously published brain organoid transcriptomes [38, 41, 62, 63] (Figure 3E). We found that brain organoids after periodically heating suggested a close resemblance with ASD organoids induced by valproic acid (VPA). Therefore, we further compared DEGs and the ASD risk genes of SFARI, and identified potential genetic alterations associated with ASD that might be related to periodically heating (Figure S3C,D).

In summary, we delved into the ramifications of periodic thermal exposure on the transcriptomic alterations of brain organoids, with a specific focus on unravelling the consequential modulations in genes related to NDDs that might be instigated by such heating exposure. Future studies should further investigate the relationship between NDDs and genetic changes induced by periodically heating.

### 3.4 Periodic Heat Exposure Led to Decreased Proliferation and Increased Apoptosis of Neural Stem Cells in Brain Organoids

**FIGURE 4:**
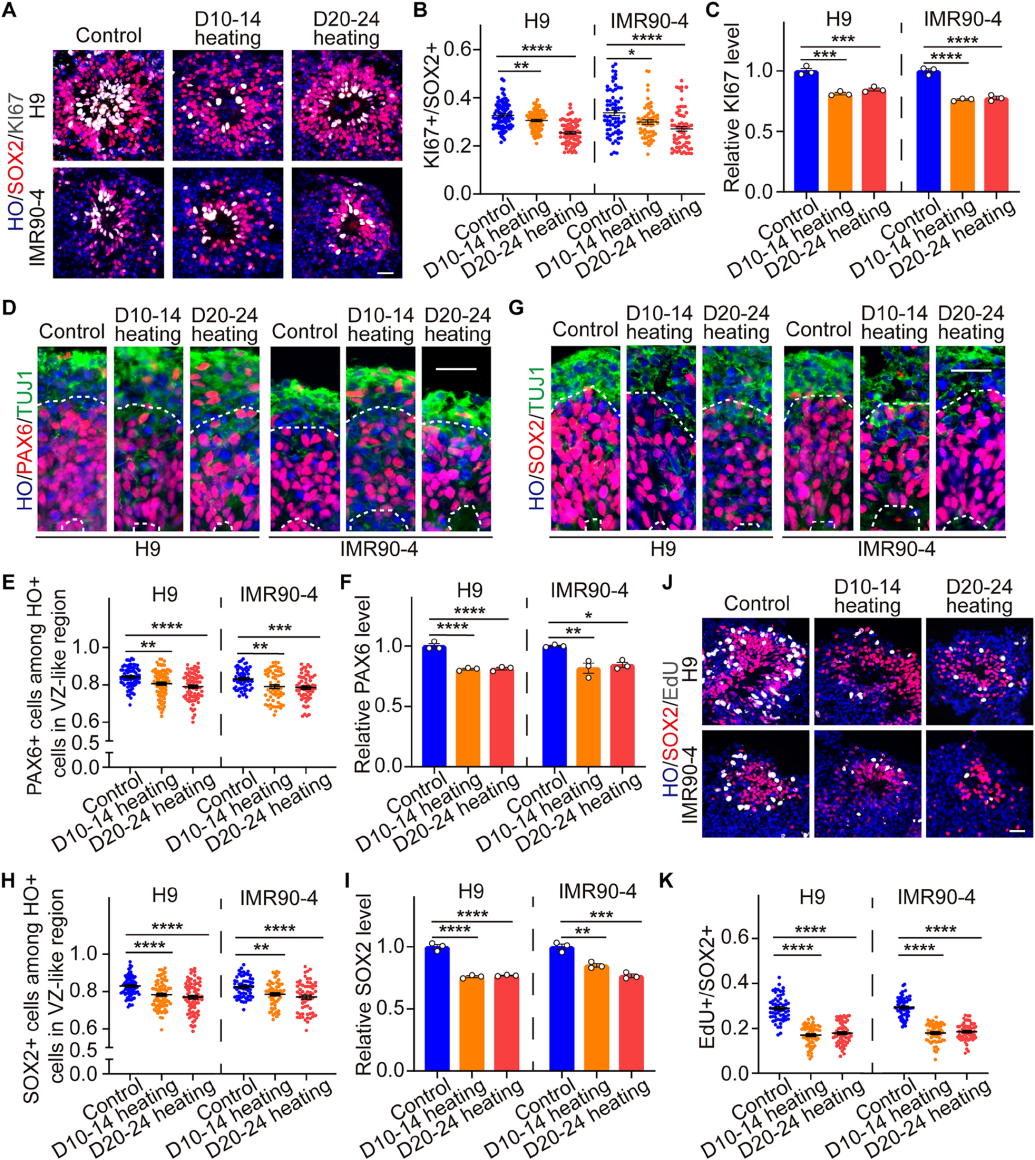
Brain organoids reduced proliferation after periodically heating at day 30. (A, B) Representative immunofluorescent staining and the proportion of KI67+ proliferative cells relative to SOX2+ neural progenitor cells within brain organoids derived from ESCs and iPSCs in three groups of the D10‐14 heating, D20‐24 heating and control at day 30 (H9, Control: *n* = 86, D10‐14 heating: *n* = 79, D20‐24 heating: *n* = 75; IMR90‐4, Control: *n* = 70, D10‐14 heating: *n* = 61, D20‐24 heating: *n* = 64). Scale bars, 50 μm. (C) Relative KI67 levels of brain organoids in three groups detected by qPCR. (D, E) Representative immunofluorescent staining and the positive rate of PAX6 in VZ‐like region within brain organoids in three groups of the D10‐14 heating, D20‐24 heating and control at day 30 (H9, Control: *n* = 74, D10‐14 heating: *n* = 86, D20‐24 heating: *n* = 80; IMR90‐4, Control: *n* = 60, D10‐14 heating: *n* = 60, D20‐24 heating: *n* = 60). Scale bars, 50 μm. (F) Relative PAX6 levels of brain organoids in three groups detected by qPCR. (G, H) Representative immunofluorescent staining and the positive rate of SOX2 in VZ‐like region within brain organoids in three groups of the D10‐14 heating, D20‐24 heating and control at day 30 (H9, Control: *n* = 81, D10‐14 heating: *n* = 78, D20‐24 heating: *n* = 80; IMR90‐4, Control: *n* = 60, D10‐14 heating: *n* = 60, D20‐24 heating: *n* = 60). Scale bars, 50 μm. (I) Relative SOX2 levels of brain organoids in three groups detected by qPCR. (J, K) Representative immunofluorescent staining and the proportion of EdU+ cells relative to SOX2+ cells within brain organoids in three groups at day 30 (H9, Control: *n* = 60, D10‐14 heating: *n* = 60, D20‐24 heating: *n* = 60; IMR90‐4, Control: *n* = 60, D10‐14 heating: *n* = 60, D20‐24 heating: *n* = 60). Scale bars, 50 μm. Each data point and relative error bar corresponds to the average and standard deviation of at least three measured samples for at least three independent experiments. Statistical analysis was performed by ANOVA. *p < 0.05, **p < 0.01, ***p < 0.001, ****p < 0.0001.

We found that heat exposure induced a reduction in the size of brain organoids (Figure 1), and the decreased proliferation after heat exposure might be a potential cause. Periodic thermal exposure appeared to attenuate cellular proliferation in brain organoids, as suggested by Bulk RNA‐seq analyses (Figure 3B). To affirm this observation, we assessed the proliferation marker KI67 in six groups of brain organoids at day 30 by immunofluorescence staining (H9, Control: 32.75% ± 5.49%, D10‐14 heating: 30.55% ± 4.04%, D20‐24 heating: 25.42% ± 4.71%; IMR90‐4, Control: 33.77% ± 9.25%, D10‐14 heating: 29.87% ± 6.91%, D20‐24 heating: 27.07% ± 8.21%) (Figure 4A), which revealed a discernible reduction in the positivity rate of KI67 after periodically heating (Figure 4B). The finding was corroborated by qPCR, which confirmed diminished transcript levels of KI67 across six groups of organoids (Figure 4C).

Given the observed decrease in total loop area of the brain organoids due to periodic heat exposure (Figure 2D), we determined the expression of VZ markers PAX6 (H9, Control: 84.14% ± 5.55%, D10‐14 heating: 80.66% ± 7.32%, D20‐24 heating: 79.00% ± 7.11%; IMR90‐4, Control: 83.14% ± 5.24%, D10‐14 heating: 79.02% ± 8.01%, D20‐24 heating: 78.51% ± 6.98%) and SOX2 (H9, Control: 84.14% ± 5.24%, D10‐14 heating: 80.66% ± 7.22%, D20‐24 heating: 79.00% ± 8.18%; IMR90‐4, Control: 83.14% ± 5.45%, D10‐14 heating: 79.02% ± 6.54%, D20‐24 heating: 78.51% ± 8.19%). The results indicated a consistent reduction in the positive rates of PAX6 and SOX2 among the organoids after D10‐14 and D20‐24 heating (Figure 4D,E,G,H), which was again substantiated by qPCR (Figure 4F,I). These findings also suggested that periodic heat exposure impaired the proliferative capacity within the VZ‐like region of brain organoids.

To further validate these findings, we incubated six groups of organoids with EdU for 2 h at day 30, followed immediately by sample collection for proliferation assessment. The ensuing results highlighted a decrement in the cycling cells within the VZ‐like region (H9, Control: 28.90% ± 5.52%, D10‐14 heating: 16.94% ± 4.52%, D20‐24 heating: 17.81% ± 4.85%; IMR90‐4, Control: 29.31% ± 4.33%, D10‐14 heating: 17.85% ± 4.12%, D20‐24 heating: 18.46% ± 3.79%), indicating a suppressive effect of periodic heat exposure on the proliferative capacity of brain organoids (Figure 4J,K).

In addition to reduced proliferation, increased apoptosis might also contribute to the decrease in the size of brain organoids, and the GSEA results of Bulk RNA‐seq also showed an increase in apoptosis of brain organoids after periodic heat exposure (Figures 3B and S2). Therefore, we further utilised the TUNEL assay to detect the effect of periodic heat exposure on apoptosis in brain organoids at day 30. The results showed that periodic heat exposure significantly increased apoptosis of brain organoids at day 30 (H9, Control: 0.20 ± 0.02, D10‐14 heating: 0.33 ± 0.03, D20‐24 heating: 0.39 ± 0.03; IMR90‐4, Control: 0.24 ± 0.02, D10‐14 heating: 0.38 ± 0.02, D20‐24 heating: 0.41 ± 0.02) (Figure S4).

In conclusion, our comprehensive analysis demonstrated that periodic thermal exposure significantly induced the reduced proliferation and increased apoptosis of brain organoids, providing a potential explanation for the reduction in their size.

### 3.5 Enhanced Neural Differentiation and Abnormal Division Dynamics in Brain Organoids Were Induced by Periodically Heating

**FIGURE 5:**
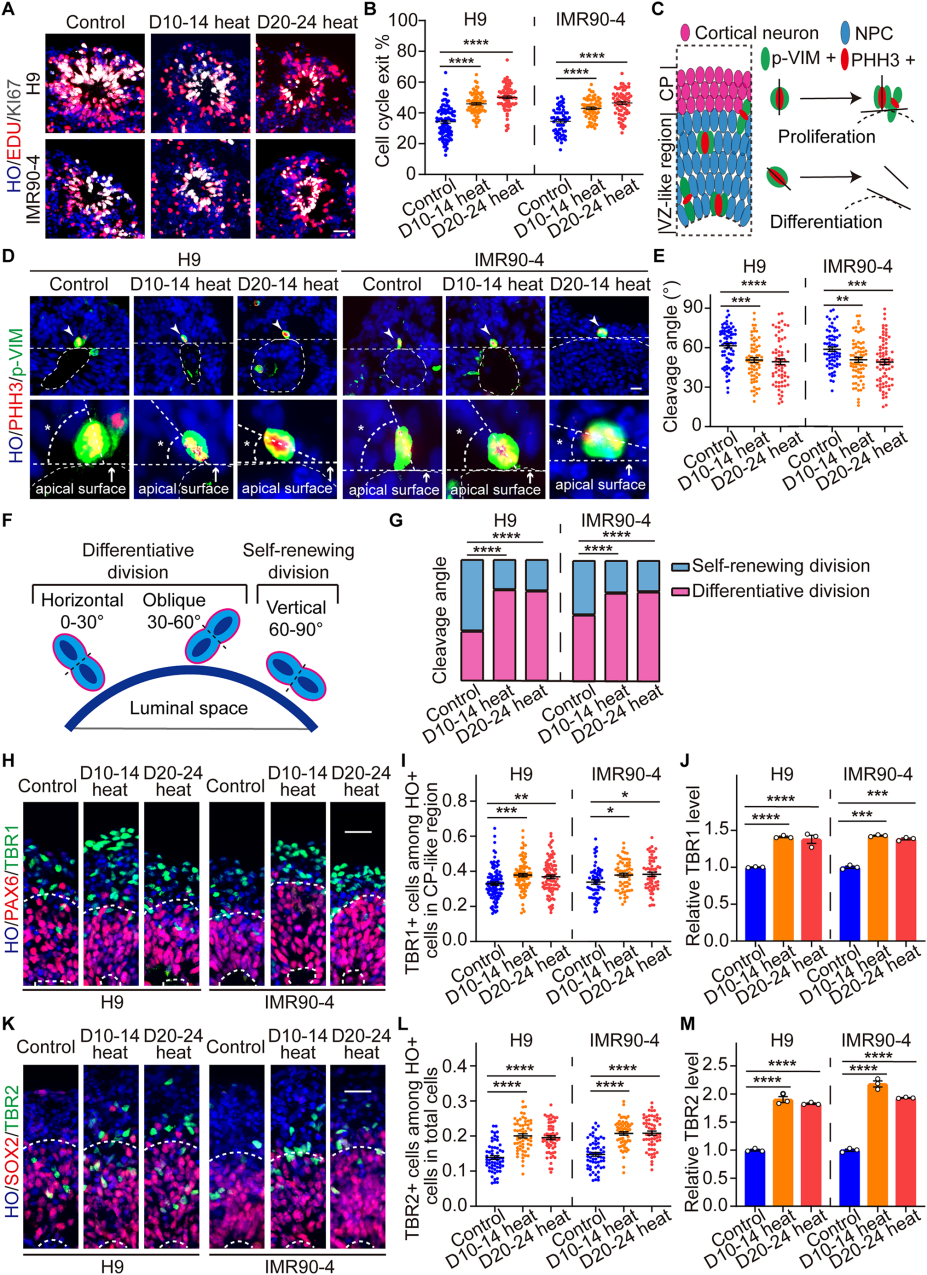
Brain organoids increased neural differentiation after periodically heating at day 30. (A, B) Representative immunofluorescent staining and the percentage of exiting the cell cycle within brain organoids derived from ESCs and iPSCs in three groups of the D10‐14 heating, D20‐24 heating and control at day 30 (H9, Control: *n* = 97, D10‐14 heating: *n* = 70, D20‐24 heating: *n* = 80; IMR90‐4, Control: *n* = 58, D10‐14 heating: *n* = 64, D20‐24 heating: *n* = 67). Scale bars, 50 μm. (C) Schematic diagram of cleavage angle marked by PHH3 and p‐VIM. (D, E) Representative immunofluorescent staining and the cleavage angle of brain organoids in three groups at day 30 (H9, Control: *n* = 73, D10‐14 heating: *n* = 67, D20‐24 heating: *n* = 65; IMR90‐4, Control: *n* = 66, D10‐14 heating: *n* = 71, D20‐24 heating: *n* = 74). Scale bars, 50 μm. (F) Schematic diagram of self‐renewing and differentiative division. (G) Stacked histogram of self‐renewing and differentiative division in brain organoids in three groups. (H, I) Representative immunofluorescent staining and the positive rate of TBR1 in CP‐like region within brain organoids in three groups of the D10‐14 heating, D20‐24 heating and control at day 30 (H9, Control: *n* = 103, D10‐14 heating: *n* = 90, D20‐24 heating: *n* = 90; IMR90‐4, Control: *n* = 60, D10‐14 heating: *n* = 60, D20‐24 heating: *n* = 60). Scale bars, 50 μm. (J) Relative TBR1 levels of brain organoids in three groups detected by qPCR. (K, L) Representative immunofluorescent staining and the positive rate of TBR2 in brain organoids in three groups of the D10‐14 heating, D20‐24 heating and control at day 30 (H9, Control: *n* = 60, D10‐14 heating: *n* = 60, D20‐24 heating: *n* = 60; IMR90‐4, Control: *n* = 60, D10‐14 heating: *n* = 60, D20‐24 heating: *n* = 60). Scale bars, 50 μm. (M) Relative TBR2 levels of brain organoids in three groups detected by qPCR. Each data point and relative error bar corresponds to the average and standard deviation of at least 60 measured samples for at least three independent experiments. Statistical analysis was performed by ANOVA or Chi‐square test. *p < 0.05, **p < 0.01, ***p < 0.001, ****p < 0.0001.

Based on the up‐regulated transcription factors of neural differentiation in Bulk RNA‐seq results (Figure 3B), we further analysed the effect of periodic heat exposure on neural differentiation within brain organoids. Utilising EdU to detect neural differentiation of brain organoids, we observed organoids following a two‐hour EdU pulse at day 28, succeeded by a medium refresh and an additional 48‐h continuous culture. Cells labelled as EdU+ KI67+ were identified as still in the proliferative phase, whereas those labelled as EdU+ KI67− were characterised as having exited the cell cycle. We quantified the proportion of cells exiting the cell cycle by assessing the ratio of EdU+ KI67− relative to the total EdU‐stained cell population. Statistical data revealed that more cells in brain organoids exit the cell cycle after periodically heating (H9, Control: 32.67% ± 9.99%, D10‐14 heating: 45.91% ± 6.82%, D20‐24 heating: 49.52% ± 7.97%; IMR90‐4, Control: 34.58% ± 8.46%, D10‐14 heating: 42.97% ± 6.65%, D20‐24 heating: 46.45% ± 8.46%), suggestive of an accelerated differentiation trajectory (Figure 5A,B).

Subsequent immunofluorescent staining for PHH3 and p‐VIM provided insights into the cleavage angles during organoid cell division (Figure 5C,D). Correlating cleavage angles with differentiation status, we found that periodic heat exposure reduced the cleavage angles of brain organoids (Figure 5D,E). Cells dividing within an angular range of 0°–60° are associated with differentiative division, whereas those within 60°–90° are indicative of self‐renewing division [64] (Figure 5F). Consistent with the higher instances of cells withdrawing from the cycle (Figure 5A,B), results from the chi‐square test substantiated a significant transition toward differentiative division after periodically heating, concurrently resulting in fewer cells exhibiting self‐renewal (Figure 5G). Such changes in proliferation and differentiation dynamics might offer a potential mechanistic explanation for the disrupted cortical stratification and the alterations in cell type elucidated in our sequencing analysis (Figure 3B).

To further substantiate the effect of thermal stimulation on neural differentiation, we assessed the biomarkers of deep layers TBR1 and the biomarkers of subplate TBR2 in six groups of organoids at day 30 by immunofluorescence and qPCR (Figure 5H–M). Our findings revealed an upregulation of both TBR1 (H9, Control: 32.92% ± 9.07%, D10‐14 heating: 37.84% ± 8.97%, D20‐24 heating: 36.88% ± 9.87%; IMR90‐4, Control: 33.82% ± 9.30%, D10‐14 heating: 37.83% ± 8.39%, D20‐24 heating: 38.30% ± 9.29%) and TBR2 (H9, Control: 13.89% ± 4.09%, D10‐14 heating: 20.06% ± 4.47%, D20‐24 heating: 19.54% ± 4.34%; IMR90‐4, Control: 14.79% ± 4.12%, D10‐14 heating: 20.75% ± 3.95%, D20‐24 heating: 20.82% ± 4.53%) in brain organoids post‐thermal treatment, thereby indicating that alterations in proliferation and differentiation dynamics indeed promoted neural differentiation within these brain organoids. Taken together, these findings highlight the significant influence of thermal stress on the biological processes in the development of brain organoids.

## 4 Discussion

The development of the human foetal brain is sensitive to environmental stimuli, particularly thermal stimuli, which disrupts numerous facets of foetal developmental processes and cerebral function [65]. Foetal NDDs including microcephaly and NTDs may have associations with maternal heat stress during gestation [2, 7, 8, 9, 10, 11, 12, 56, 57, 58, 59, 60]. Nonetheless, the absence of in vitro models replicating human cerebral development has hampered advancements in elucidating the impacts of heat stress on the development of the human brain. As an in vitro human brain model, brain organoids derived from hPSCs could be used to explore the phenotypes and mechanisms of environmental stimuli on brain development [32, 33, 34]. Particularly in light of the global COVID‐19 pandemic in recent years, the significance of brain organoids as a tool for investigating the influence of maternal fever on foetal brain development could be markedly amplified [27].

In our study, we presented an in vitro exploration into the effects of hyperthermia on brain organoids derived from hPSCs, modelling the impact of maternal fever during early pregnancy on foetal brain development. The introduction of a periodic heat exposure scheme at 40°C, designed to model febrile episodes, furnishes a method to study thermal damage. The observed diminution in the size of the organoids post‐heat treatment was consistent with the possible foetal microcephaly observed in animal models or clinical studies after maternal thermal damage during pregnancy [12, 15, 16, 17], suggesting potential for the model to mimic aspects of foetal microcephaly and other NDDs induced by thermal stress. Our results indicated that the reduction in the size of brain organoids after heat exposure might be attributed to decreased proliferation and increased apoptosis, which was consistent with the observations in animal studies [14, 15, 19]. Although clinical data remains limited, the findings from brain organoids might provide supportive evidence for the mechanism.

Our findings also suggested that the total loop area of brain organoids decreased after heat exposure, revealing that thermal stimulation might lead to abnormal neural tube development. This finding might be related to the observations of hyperthermia‐induced NTDs in clinical and animal studies [2, 7, 8, 9, 10, 11, 20, 21]. The disruption of rosette formation might be attributed to the heat‐induced disruption of cell membranes during neural tube closure [2]. Notably, D20‐24 heat exposure did not modify the ventricle area of brain organoids, while D10‐14 heat exposure resulted in a notable reduction of this area. The results indicated that the apical surface is more vulnerable to thermal damage during the neural tube formation stage. The implications of these findings are particularly relevant for investigating the teratogenic effects induced by hyperthermia during embryonic neural development. The underlying mechanisms appeared to involve a notable shift in the dynamics of cellular proliferation and differentiation within the organoids. The decline in size and the impaired neural tube development signified not only a deviation from normal growth patterns but also highlight a vulnerability of the developing neural tissue to environmental stress.

The bulk RNA‐Seq analysis further elucidated transcriptomic features marked by the upregulation of WNT signalling pathway and neural differentiation transcription factors, accompanying a downregulation of G2/M progenitors. These findings underscored a complex interplay between thermal stress and genetic regulation, potentially steering the organoids toward premature differentiation and altered development trajectories. Furthermore, cross‐species studies have shown that burn injury or hyperthermia could lead to the upregulation of WNT signalling, potentially triggered by some genes related to heat adaptation following hyperthermia [66, 67, 68, 69]. It was worth noting that WNT5A emerged as a pivotal gene within PPI analysis. WNT5A is instrumental in activating the WNT/PCP signalling pathway, thereby influencing neural tube closure by convergence extension [51, 52, 53, 54, 55]. This process potentially constituted one of the underlying factors contributing to the manifestation of NTDs.

Moreover, the research delineated the utility of brain organoids as a versatile and predictive model for assessing the teratogenic impact of environmental factors on human neurodevelopment. We compared sequencing data with multiple databases to screen for a series of genetic changes caused by heat stress that might affect NDDs including ASD. Notably, brain organoids after periodically heating were closest in transcriptome to ASD brain organoids induced by VPA, and studies on VPA‐induced ASD brain organoids had also shown similar reductions in size and proliferation, and abnormal neural tube development as those after periodically heating [49, 70]. It might reveal the contribution of thermal stress in mediating NDDs, providing insights that could be pivotal for devising therapeutic strategies and protective measures against such perturbations in early pregnancy. Additionally, brain organoids could be used for high‐throughput drug screening and the validation of therapeutic interventions, such as timely cooling for heat exposure in early pregnancy.

In summary, our study elucidated the adverse impacts of hyperthermia during early pregnancy on foetal brain development through the use of human brain organoids. We demonstrated that periodic heat exposure could significantly reduce the size of organoids, impair neural tube development and alter gene expression pathways, notably the WNT signalling pathway. These findings suggested a mechanism by which maternal hyperthermia might contribute to NDDs, highlighting the potential of brain organoids as a model for understanding the effects of environmental stimulation on foetal brain development.

## Author Contributions

L. Xu, M. Xu, W. Zhu and Y. Liu designed the study. L. Xu, Y. Zhang, X. Chen, H. Hu, X. Zhang and X. Zou performed the experiments. L. Xu, Y. Zhang and X. Han analysed the data. L. Xu, X. Zhang and Y. Hong were responsible for sequencing analysis. W. Zhu, M. Xu and Y. Liu supervised the work. L. Xu and Y. Liu wrote the manuscript. All authors read and approved the final manuscript.

## Ethics Statement

All cell lines in this article are from WiCell, ethical approval is not applicable to this article. This article does not contain any studies with humans or animals performed by any of the authors.

## Conflicts of Interest

The authors declare no conflicts of interest.

## Supporting information


**Figure S1.** Bulk RNA‐seq transcriptional profiling of brain organoids derived from ESCs in three groups of the D10‐14 heating, D20‐24 heating and control at day 30. (A) PCA showing the differentiation between the D10‐14 heating, D20‐24 heating organoids and the control. Each group of brain organoids contains three replicates, and each replicate contains at least five brain organoids. (B–D) The volcano map and the histogram showing up‐regulated and down‐regulated genes found in brain organoids after periodic heating compared with the control. (E) Venn’s diagrams drawing 140 overlapping DEGs that up‐regulated due to D10‐14 and D20‐24 heating. (F) GO analysis of biological process, cellular component and molecular function for 140 overlapping DEGs. Presented GO terms are all significantly changed (adjusted *p*‐value < 0.05). (G) KEGG analysis for 140 overlapping DEGs. (H) PPI network maps showing up‐regulated WNT signalling pathway. (I) GO analysis and KEGG analysis for other non‐overlapping DEGs.
**Figure S2.** GSEA results showed the alterations in brain organoid biological pathways after periodic thermal damage.
**Figure S3.** A set of genes based on DEGs and databases was screened. (A, B) Venn’s diagrams and heatmap drawing a set of NDDs risk genes in brain organoids after periodic thermal damage. (C, D) Venn’s diagrams and heatmap drawing a set of ASD risk genes in brain organoids after periodic thermal damage.
**Figure S4.** Brain organoids increased apoptosis after periodically heating at day 30. (A, B) Representative immunofluorescent staining and the rate of TUNEL+ cells in brain organoids derived from ESCs and iPSCs in three groups of the D10‐14 heating, D20‐24 heating and control at day 30 (H9, Control: *n* = 26, D10‐14 heating: *n* = 30, D20‐24 heating: *n* = 28; IMR90‐4, Control: *n* = 29, D10‐14 heating: *n* = 26, D20‐24 heating: *n* = 24). Scale bars, 50 μm. Each data point and relative error bar corresponds to the average and standard deviation of at least three measured samples for at least three independent experiments. Statistical analysis was performed by ANOVA. ****p < 0.0001.

## Data Availability

The data that supports the findings of this study are available in the Supporting Information of this article or from the corresponding author upon reasonable request.
