# Supplementary material for: Human Brain Organoids Model Abnormal Prenatal Neural Development Induced by Thermal Stimulation
Source: Cell Prolif. 2024 Dec 12;58(2):e13777. doi: 10.1111/cpr.13777 (PMC11839188; doi:10.1111/cpr.13777)
Supplement: Supplementary file 1 — Figure S1. Bulk RNA‐seq transcriptional profiling of brain organoids derived from ESCs in three groups of the D10‐14 heating, D20‐24 heating and control at day 30. (A) PCA showing the differentiation between the D10‐14 heating, D20‐24 heating organoids and the control. Each group of brain organoids contains three replicates, and each replicate contains at least five brain organoids. (B–D) The volcano map and the histogram showing up‐regulated and down‐regulated genes found in brain organoids after periodic heating compared with the control. (E) Venn’s diagrams drawing 140 overlapping DEGs that up‐regulated due to D10‐14 and D20‐24 heating. (F) GO analysis of biological process, cellular component and molecular function for 140 overlapping DEGs. Presented GO terms are all significantly changed (adjusted p‐value < 0.05). (G) KEGG analysis for 140 overlapping DEGs. (H) PPI network maps showing up‐regulated WNT signalling pathway. (I) GO analysis and KEGG analysis for other non‐overlapping DEGs. Figure S2. GSEA results showed the alterations in brain organoid biological pathways after periodic thermal damage. Figure S3. A set of genes based on DEGs and databases was screened. (A, B) Venn’s diagrams and heatmap drawing a set of NDDs risk genes in brain organoids after periodic thermal damage. (C, D) Venn’s diagrams and heatmap drawing a set of ASD risk genes in brain organoids after periodic thermal damage. Figure S4. Brain organoids increased apoptosis after periodically heating at day 30. (A, B) Representative immunofluorescent staining and the rate of TUNEL+ cells in brain organoids derived from ESCs and iPSCs in three groups of the D10‐14 heating, D20‐24 heating and control at day 30 (H9, Control: n = 26, D10‐14 heating: n = 30, D20‐24 heating: n = 28; IMR90‐4, Control: n = 29, D10‐14 heating: n = 26, D20‐24 heating: n = 24). Scale bars, 50 μm. Each data point and relative error bar corresponds to the average and standard deviation of at least three measured samples [file CPR-58-e13777-s001.docx]

**Supplementary Information**

**Periodically Heated Brain Organoids Model Abnormal Human Brain Development Induced by Prenatal Heat Exposure**

Lei Xu^1, 2, 3^, Yufan Zhang^2, 3^, Xingyi Chen^2, 3^, Yuan Hong^2, 3^, Xu Zhang^2, 3^, Hao Hu^2, 3^, Xiao Han^2, 3^, Xiao Zou^2^, Min Xu^2, 3^*, Wanying Zhu^2, 3^*, Yan Liu^1, 2, 3^*

^1^State Key Laboratory of Digital Medical Engineering, School of Biological Science and Medical Engineering; Department of neurology, affiliated Zhongda Hospital, Southeast University, Nanjing, 210096, China

^2^Institute of Stem Cell and Neural Regeneration, School of pharmacy, Nanjing Medical University, Nanjing, 211166, China

^3^State Key Laboratory of Reproductive Medicine and Offspring Health, Nanjing Medical University, Nanjing, 211166, China

**Supplemental Figures**


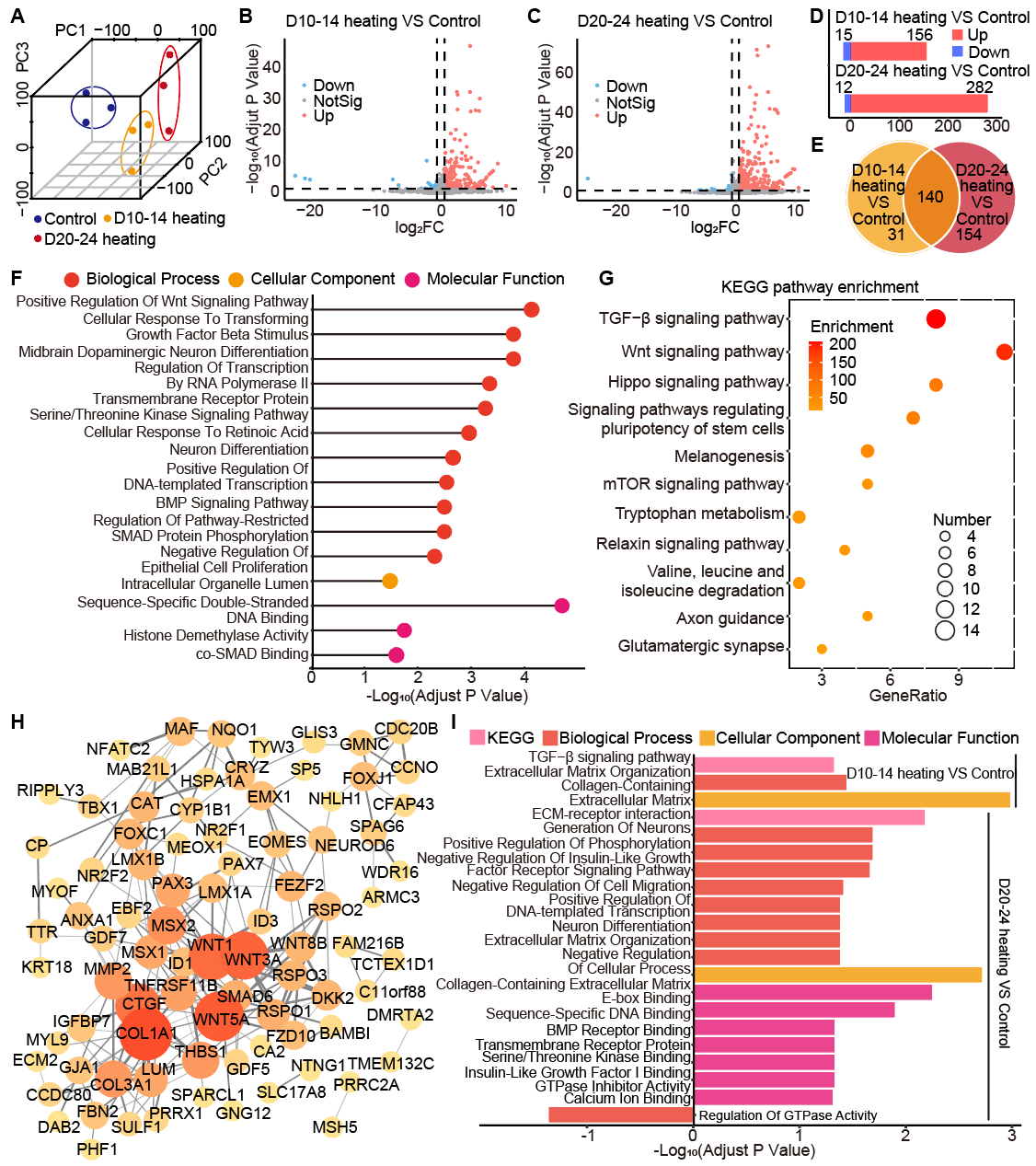


**Figure S1.** Bulk RNA-seq transcriptional profiling of brain organoids derived from ESCs in three groups of the D10-14 heating, D20-24 heating and control at day 30. (A) PCA showing the differentiation between the D10-14 heating, D20-24 heating organoids and the control. Each group of brain organoids contains three replicates, and each replicate contains at least five brain organoids. (B-D) The volcano map and the histogram showing up-regulated and down-regulated genes found in brain organoids after periodic heating compared with the control. (E) Venn's diagrams drawing 140 overlapping DEGs that up-regulated due to D10-14 and D20-24 heating. (F) GO analysis of biological process, cellular component and molecular function for 140 overlapping DEGs. Presented GO terms are all significantly changed (adjusted P‐value < 0.05). (G) KEGG analysis for 140 overlapping DEGs. (H) PPI network maps showing up-regulated WNT signaling pathway. (I) GO analysis and KEGG analysis for other non-overlapping DEGs.


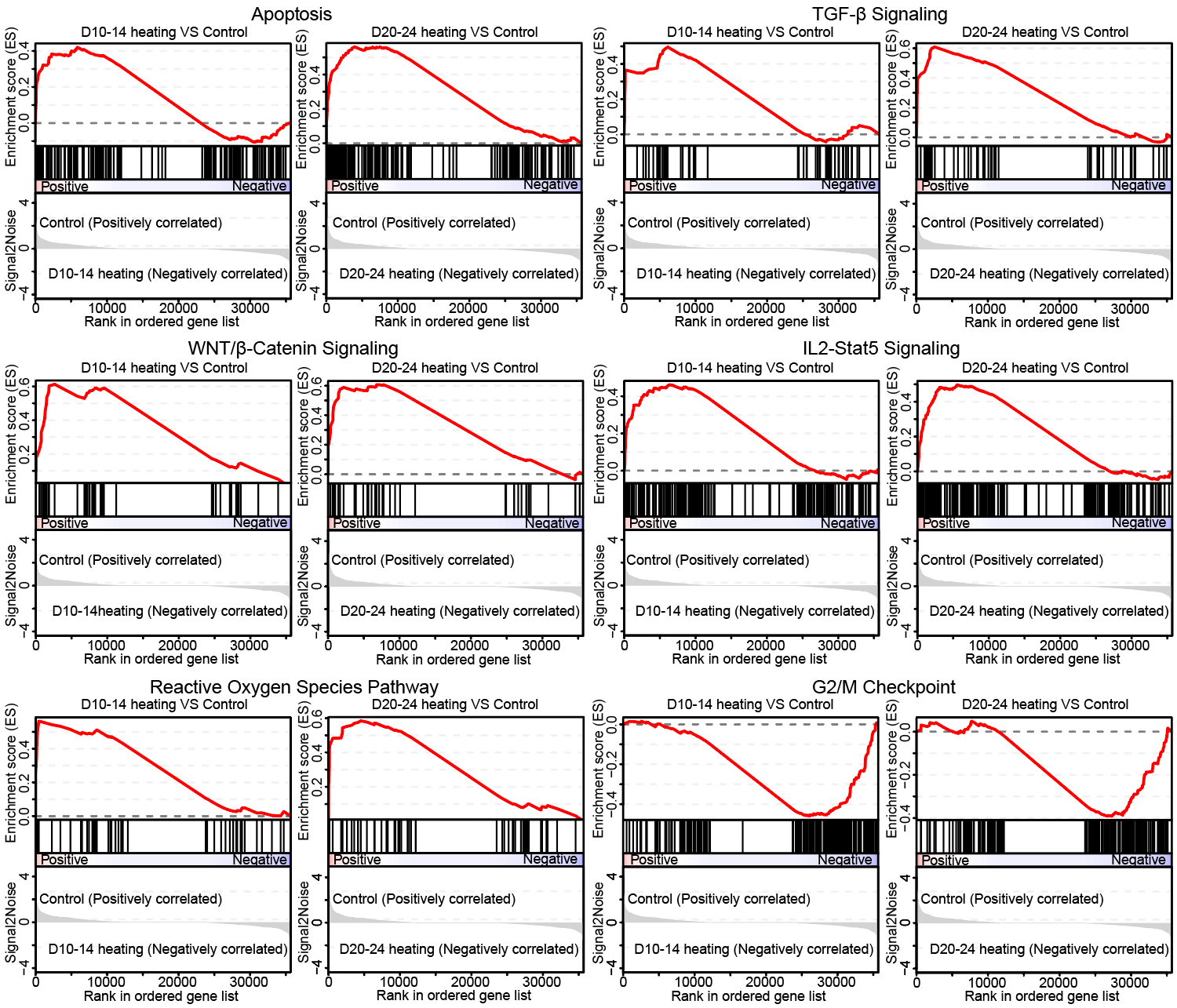


**Figure S2.** GSEA results showed the alterations in brain organoid biological pathways after periodic thermal damage.


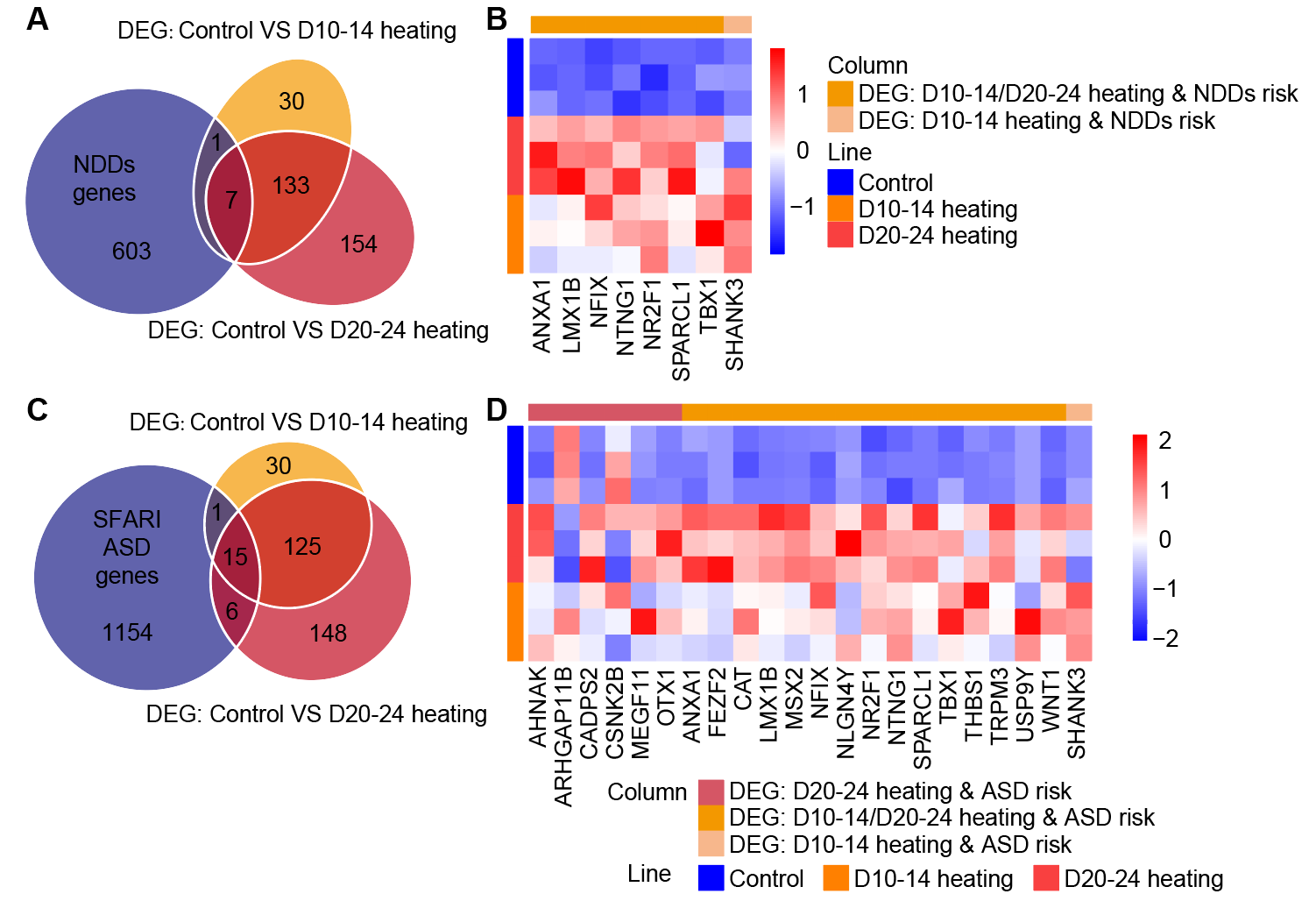


**Figure S3.** A set of genes based on DEGs and databases was screened. (A, B) Venn's diagrams and heatmap drawing a set of NDDs risk genes in brain organoids after periodic thermal damage. (C, D) Venn's diagrams and heatmap drawing a set of ASD risk genes in brain organoids after periodic thermal damage.


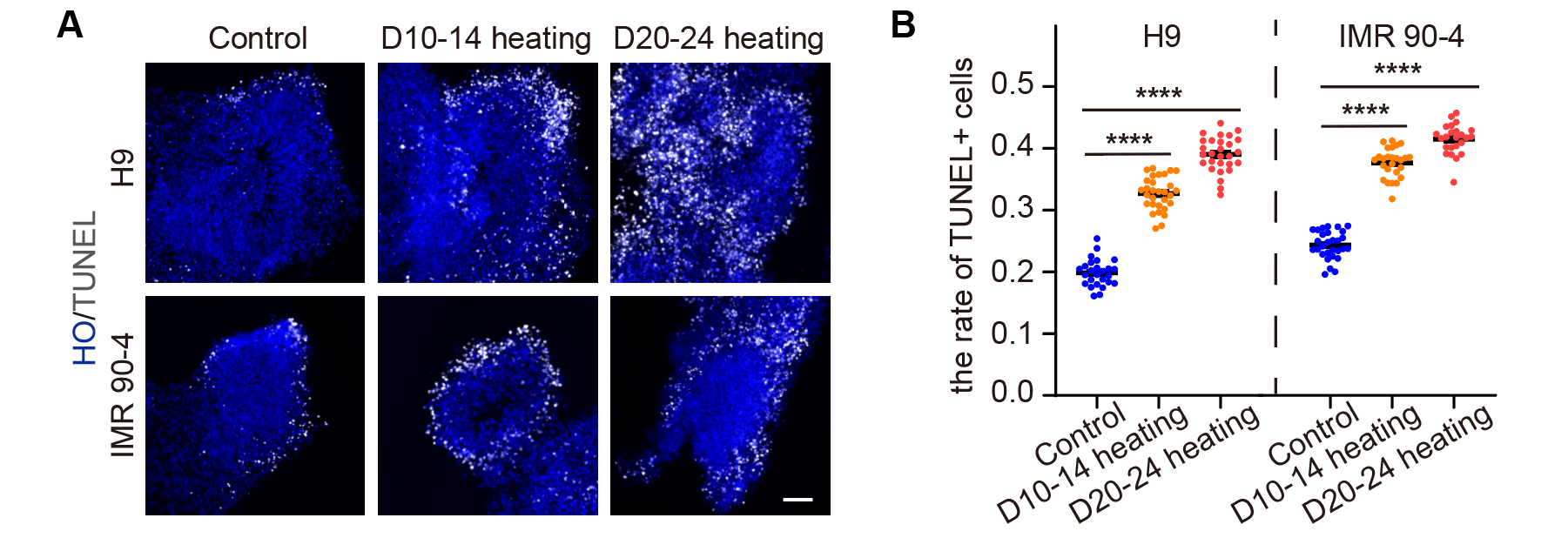


**Figure S4.** Brain organoids increased apoptosis after periodically heating at day 30. (A, B) Representative immunofluorescent staining and the rate of TUNEL+ cells in brain organoids derived from ESCs and iPSCs in three groups of the D10-14 heating, D20-24 heating and control at day 30 (H9, Control: n=26, D10-14 heating: n=30, D20-24 heating: n=28; IMR90-4, Control: n=29, D10-14 heating: n=26, D20-24 heating: n=24). Scale bars, 50 μm. Each data point and relative error bar corresponds to the average and standard deviation of at least three measured samples for at least three independent experiments. Statistical analysis was performed by ANOVA. ****p< 0.0001.
